# Customized small-sized clinostat using 3D printing and gas-permeable polydimethylsiloxane culture dish

**DOI:** 10.1038/s41526-023-00311-1

**Published:** 2023-08-11

**Authors:** Daehan Kim, Que Thanh Thanh Nguyen, Seungjin Lee, Kyung-Mi Choi, Eun-Ju Lee, Joong Yull Park

**Affiliations:** 1 Department of Mechanical Engineering, Graduate School, Chung-Ang University, Seoul, 06974 Republic of Korea; 2 Department of Obstetrics and Gynecology, School of Medicine, Chung-Ang University, Seoul, 06974 Republic of Korea; 3 Department of Intelligent Energy and Industry, Graduate School, Chung-Ang University, Seoul, 06974 Republic of Korea

**Keywords:** Biomedical engineering, Cancer prevention, Aerospace engineering

## Abstract

Over the past few decades, research on life in space has increased. Owing to the expensive nature of and the challenges associated with conducting experiments in real space, clinostats, which continuously randomize the gravity vector by using motors, have been used to generate simulated microgravity (SMG) on Earth. Herein, by using a 3D printing method, we develop a customized small-sized clinostat (CS clinostat) that is easy to manufacture, inexpensive, and robust. Moreover, we develop and fabricate a gas-permeable polydimethylsiloxane culture dish that fits inside the CS clinostat. To validate SMG generation, ovarian cancer cells (OV- 90, TOV-21G, and Caov-3) were applied to demonstrate a significant reduction in caveolin-1 expression, a biomarker of SMG, indicating SMG generation. The proposed CS clinostat system has good accessibility for SMG research, which makes it useful as a tool for biologists, who are unfamiliar with conventional clinostat equipment, to conduct preliminary studies in the space environment.

## Introduction

All living organisms on Earth, including the cells inside the human body, adjust for constant gravity. Observation reports of astronauts who have stayed in space for an extended period indicated significant body changes such as sarcopenia^1^ and osteopenia^2^. Because of these changes, it is important to understand the effect of low or zero gravity at the cellular level. Conducting experiments with zero gravity in space would be ideal. However, owing to time and resource constraints, systems such as magnetic-field-induced levitation^3^, rotating-wall vessels (RWVs)^4^, and clinostats^5^ have been proposed to simulate weightlessness on Earth. The range of real microgravity, such as that in spacecraft or on the International Space Station (ISS), is ~10^−3^ to 10^−6^ g ^6–8^. Although these systems that simulate weightlessness on Earth cannot generate an actual microgravity environment as occurs in freefall, they can provide a time-averaged net displacement equivalent to an approximately 10^−2^ to 10^−5^ g gravity environment, which is called simulated microgravity (SMG)^9,10^.

**Fig. 1:**
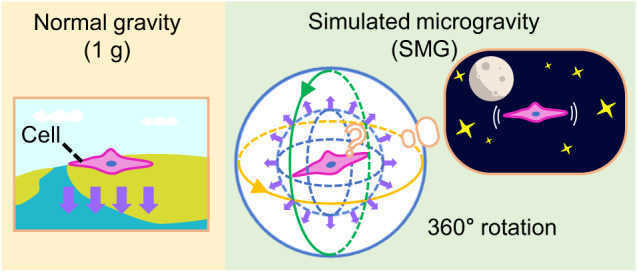
Principle for generating simulated microgravity (SMG) environment using the three-dimensional rotation of a clinostat. Cells on Earth are cultured in normal gravity (1 g) environment with constant gravity along the −z direction. However, because the cells are rotated in a clinostat, the direction of gravity is constantly reoriented, and the cells experience SMG.

The clinostat, a device that simulates microgravity through rotational motion, was first proposed in the early 1900s and has since been used actively in plant and cell experiments^11–13^. Clinostats are the most commonly used system in SMG research because they can effectively create an SMG environment. Clinostats can be categorized into two-dimensional (2D) clinostats that rotate about the horizontal axis by using a single motor, three-dimensional (3D) clinostats that rotate about two orthogonal axes by using two motors, and random positioning machines (RPMs) in which two axes rotate randomly. The most commercialized clinostat-type system is RWVs (models RCCS-2D, RCCS-1, etc., from Synthecon Inc.), which are based on the same mechanism as 2D clinostats that rotate about one axis. However, while 2D clinostats can be used to culture adherent cells at low speeds (<10 revolutions per minute (rpm)), RWVs are used for suspension culture with floating cells at high speeds of 10–60 rpm and cannot achieve 3D rotation similar to a 3D clinostat. In principle, a clinostat simulates microgravity by continuously reorienting the gravity vector acting on any object inside it (Fig. 1)^14^. Inside a clinostat, the gravity applied to a constantly rotating object is always directed along the −z direction, but because the inner object rotates, gravity eventually acts on the object along all directions. Consequently, the average gravity load is ~1 at each instant. Nevertheless, because the direction of gravity is changing constantly, the average gravity direction is closer to 0 over time. Biological systems inside a clinostat, such as cells, continue to experience Earth’s gravity. However, the direction of this gravity changes constantly, and therefore, the systems are in an SMG environment that is different from a normal gravity environment^13,15^.

Studies have reported various effects of SMG on mammalian cells. Human umbilical vein endothelial cells (HUVECs) cultured in a 2D clinostat exhibited decreased caveolin-1 (CAV-1) protein expression and increased endothelial nitric oxide synthase (eNOS) expression in the SMG environment^16^. When the human Hodgkin’s lymphoma cell lines L-540 and HDLM- 2 were exposed to the SMG environment, cell proliferation decreased. By contrast, human dermal fibroblast (HDF) cells exhibited no significant change in cell proliferation^10^. In the cases of the human carcinoma cell lines FTC-133 and CGTH W-1, the expressions of vimentin and tubulin β proteins increased in FTC-133 after 3 days of clinorotation, whereas the expressions of the same proteins decreased in CGTH W-1, and the expressions of actin protein decreased in both cell lines^17^. Because these previous experiments were conducted using different cell lines and under diverse conditions, including 2D clinostat, 3D clinostat, different rotation speeds, and different cell lines, their outcomes may have differed accordingly. Therefore, standardized clinostat-based testing procedures and metrics should be established.

The existing clinostat devices are unsuitable for biologists because of their large sizes and hard constituent materials. For instance, the existing clinostats do not fit into the typical 50 L incubators used in laboratories. Moreover, the large cell culture space within the existing clinostats does not produce a uniform SMG environment owing to considerable variations in the centrifugal and tangential accelerations exerted on the cells proportional to their distance from the center of rotation^13^. In addition, the use of aluminum or steel as clinostat materials warrants the use of specific manufacturing expertise. These factors may serve as barriers for researchers from diverse fields who wish to conduct SMG studies. Therefore, a clinostat that can fit inside a general incubator, be manufactured and handled easily, and generate an SMG environment effectively is required.

Another important issue that should not be overlooked pertains to the culture dish. As it stands, researchers use commercially available dishes such as Petri dishes or T-flasks to conduct clinostat experiments. However, these commercial dishes are not optimized for clinostats (especially for the rotating motion) and have the following disadvantages. First, it is difficult to prevent the formation of air bubbles, which can induce unwanted mechanical stresses in the cells placed in the rotating culture dish^13^. Second, it is not easy to tightly fasten commercially available dishes to the clinostat because even extremely tiny gaps can cause undesired rocking motions. One remedy is to use tape to wrap and secure the dish to the clinostat^18^. Third, commercially available dishes are made of plastic, usually polystyrene or polycarbonate, which does not allow for gas exchange. This isolates the cells from the air inside the incubator, which is an unfavorable culture condition, especially for a long-term culture. Therefore, optimized designs of the culture dish to be used in clinostat systems are needed to compensate for the abovementioned disadvantages.

In this study, a customized small-sized clinostat (CS clinostat) that can reduce the barriers to SMG research is proposed. The proposed CS clinostat is easy to manufacture, inexpensive, and robust, and we fabricate it using a 3D printer. Moreover, because of the small size of the CS clinostat, it can be operated inside a small incubator, and because the rotation radius of the inner cell is small, the centrifugal acceleration applied to cells is minimized. Furthermore, a culture dish that fits the CS clinostat and is covered with polydimethylsiloxane (PDMS) to allow for gas exchange during cell culture is fabricated. Six PDMS clinostat dishes can be easily inserted in the CS clinostat to ensure that a large number of cells can be cultured at once. To verify the SMG environment of the CS clinostat, we mechanically measure the change in gravity inside it by using a three-axis accelerometer that measures acceleration along the *x*, *y*, and z axes from −1 g to 1 g depending on orientation. The results indicated that the mean value of the gravity vector after 1 h of operation was approximately 10^−2^ g. Moreover, the effects of SMG on the cells, including a decrease in CAV-1 expression, which is a biomarker of SMG, and viability, were observed. Furthermore, the results of tensile tests and static structural simulations indicated that the material properties of the 3D-printed CS clinostat were mechanically robust to SMG experiments. Therefore, the proposed CS clinostat is an effective SMG generator and can lower the barriers to SMG research.

## Methods

### The fabrication method of CS clinostat and PDMS clinostat dish

**Fig. 2:**
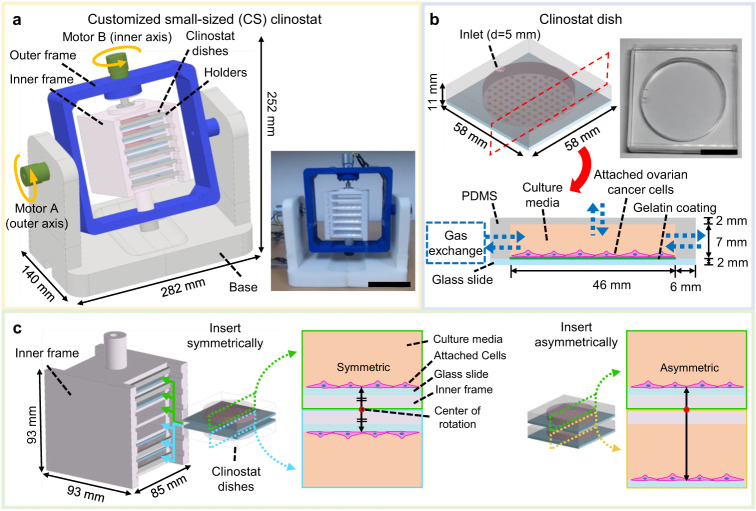
Design of CS clinostat and polydimethylsiloxane (PDMS)-based clinostat dish. **a** The CS clinostat generates a 360° three-dimensional (3D) rotation by using two motors that rotate an outer frame and an inner frame. The inner frame, outer frame, and base component are 3D-printed. Motor A, connected to the outer frame rotates at 4 rpm, and Motor B, connected to the inner frame, rotates at 1.8 rpm. The dimensions of the clinostat are 282 mm × 140 mm × 252 mm. The photograph of the CS clinostat is in an inset; the scale bar is 10 cm. **b** Schematic image and photograph of the PDMS clinostat dish. The top and side walls of the PDMS clinostat dish are made of PDMS, and the bottom is made of glass coated with gelatin. A hole with a diameter of 5 mm is located on the top surface of the PDMS clinostat dish for injecting medium and cell suspensions. The surface of the PDMS clinostat dish allows for gas exchange owing to the good permeability of PDMS; the scale bar is 20 mm. **c** Up to six PDMS clinostat dishes can be inserted inside the inner frame, the dimensions of which are 85 mm × 93 mm × 93 mm. The inserted PDMS clinostat dishes are fastened using a holder. The PDMS clinostat dishes are inserted symmetrically to achieve a symmetric culture environment. If the PDMS clinostat dishes are aligned asymmetrically, the applied acceleration force would not be symmetrical.

The CS clinostat was designed such that it can be fitted in commercial incubators, and the culture dishes were designed to be as large as possible under this size constraint to allow for the culture of a sufficient number of cells. The dimensions of the clinostat are 282 mm × 140 mm × 252 mm, and it was modeled using Autodesk Inventor 2023 (Autodesk Inc., USA) (Fig. 2a). The CS clinostat can be used in a small incubator with a capacity of 50 L (e.g., Panasonic MCO-5AC with interior dimensions of 350 mm × 378 mm × 375 mm), and the remaining space inside the incubator can be used for other experiments. The CS clinostat consists of two motors rotating about two axes, an inner frame and an outer frame. The base of the CS clinostat serves as the foundation of the overall structure, and it holds the other clinostat components. Each part of the modeled CS clinostat was converted into an STL format file, and the corresponding g-code was generated using a 3D printer software application (Cubicreator, Cubicon Co., Republic of Korea). Each component was fabricated directly by using a fused deposition modeling (FDM) 3D printer (Cubicon Style 3DP-210F, Cubicon Co., Republic of Korea). In terms of 3D printer settings, the layer height, printed outer wall thickness, and infill density were set, respectively, to 300 µm, 400 µm, and 50% (note that the typical infill density is 15–50%) (Supplementary Fig. 1). The base, outer frame, and holders were composed of acrylonitrile butadiene styrene (ABS), and the inner frame was composed of thermoplastic polyurethane (TPU). ABS, which is stronger than TPU, was selected as the material of the base and outer frame to ensure the rigidity of the entire CS clinostat system. TPU, which is relatively more elastic than ABS, was selected as the inner frame material to ensure that the PDMS clinostat dishes can be inserted easily. Brushless direct current (BLDC) gear motors with a reduction ratio of 1/1249 (BL2419A, D&J WITH Co., Republic of Korea) were used to rotate the outer and inner axes. The holding torque and rated output of these motors were 3000 gf-cm and 1.6 W, respectively. The rotational speeds of these motors were controlled using a BLDC motor controller (BLC- 22H11P-V12, D&J WITH Co., Republic of Korea). The rotational speeds of the inner frame and the outer frame were controlled to 4 rpm and 1.8 rpm, respectively, which were found to yield an effective rotation speed ratio for 3D clinostats in another study^19^. Slip rings (M220-06, Senring Electronics Co., China) were used to prevent twisting of the inner axis motor wires owing to rotation.

For the proposed CS clinostat, a customized culture dish was needed to ensure efficient experimentation without compromising cell culture because of the small internal space (Fig. 2b). During rotation, complete sealing of the dish was needed to prevent air bubble formation and media leakage. However, such complete sealing of commercially available cell culture dishes could block gas exchange and disrupt cell growth conditions. To avoid this effect, we covered the top and sides of a culture dish with PDMS, which is air-permeable, biocompatible, and transparent. A PDMS base and a PDMS curing agent (Sylgard 184, Dow Chemical Co., USA) were mixed in a ratio of 10:1 (w:w) by following the manufacturer’s instructions. The PDMS mixture was degassed for 30 min in a desiccator and then poured into an acrylic mold of the PDMS clinostat dish that was fabricated using a computerized numerical control (CNC) machine. After degassing again for 30 min, the PDMS mixture was cured in a heat chamber at 80 °C. A biopsy punch was used to create a 5-mm-diameter hole on the top surface of the cured PDMS, and this hole was used as an inlet for cells and culture media. It was possible to easily culture cells in the PDMS clinostat dish through the aforementioned inlet hole. After seeding and stabilizing the cells in the incubator under static conditions, all air bubbles were removed, holes were blocked, and the PDMS clinostat dishes were slotted in the frame for rotation. The PDMS and a sliding glass measuring 52 mm × 52 mm were exposed to air plasma (PDC-32G, Harrick Plasma Co., USA) for 30 s at a power of 18 W to bond them to each other, which completed the fabrication of the PDMS clinostat dish. The glass bottom of the PDMS clinostat dish was coated with gelatin to facilitate cell attachment. To minimize the stress produced by rotational acceleration and provide nutrition for cell growth, the cell culture area of the prepared PDMS clinostat dish was set to 46 mm in diameter and 7 mm in height.

The inner frame measuring 85 mm × 93 mm × 93 mm was designed to mount up to six PDMS clinostat dishes (Fig. 2c) in slots, and the attached holder locked the inserted dishes tightly during rotation (Supplementary Fig. 2). The clinostat dishes were inserted with the cell-attached side facing downward and upward into each of the upper and lower three slots, respectively, of the inner frame. In total, six dishes were inserted symmetrically. If the lower PDMS clinostat dishes were not inserted upside down, the alignment would be asymmetric and would lead to the application of asymmetric forces to the cells in the dishes. Therefore, the insert direction of the dishes should be selected carefully.

### Acceleration measurement using a sensor

To mechanically measure the SMG effect generated by the clinostat, an inertial measurement unit (IMU) sensor (BWT61CL, WitMotion Shenzhen Co., China) powered by a rechargeable battery and equipped with Bluetooth communication was used to measure the acceleration of the *x*–*y*–*z* axes. The data received by the smartphone application (WitMotion, WitMotion Shenzhen Co., China) were transferred to a computer, and the acceleration and mean acceleration of each axis were plotted in a graph using SigmaPlot 12 (Systat Software Inc., USA).

### Tensile tests

To ensure the mechanical stability of the CS clinostat system, bone-shaped tensile testing specimens of ABS and TPU were printed as specified in ASTM D 638 Type 4, an international standard for tensile tests. To ensure that the tensile test results reflected the actual stability of the CS clinostat system, the specimens were printed under the same conditions as those in the CS clinostat (Supplementary Fig. 1). The fabricated specimens were placed in a tension-compression tester (MCT-2150, A&D Co., Japan) and tensioned at a rate of 10 mm/min (Supplementary Fig. 3). The tensile tests were performed thrice for each material.

### Static structural simulations

To predict the mechanical stability of the CS clinostat on the basis of the mechanical properties of the 3D printer material obtained through the tensile test, a static structural simulation (ANSYS Inc., USA) of the inner frame of the CS clinostat was performed. The inner frame made of TPU was simulated, whereas the outer frame and base, both made of ABS, which has higher strength, were not simulated. The simulation was conducted using the ultimate strength and Young’s modulus obtained from the tensile test of TPU. It was assumed that the PDMS clinostat dishes, each weighing approximately 35 g, were placed in the six slots of the inner frame, and a total downward force of 2.058 N was applied. Both ends of the inner frame were assumed to be fixed. The mesh used in the analysis consisted of 7256 tetrahedral elements.

### Cell lines and culture

To prove SMG generation, mammalian cells were cultured using the CS clinostat. The TOV-21G (catalog # CRL-11730, American Type Culture Collection Co. (ATCC), USA) and OV-90 (catalog # CRL-11732, ATCC Co., USA) ovarian cancer cell lines were purchased from the ATCC, and the Caov-3 ovarian cancer cell line (catalog # 30075, Korean Cell Line Bank Co., Republic of Korea) was purchased from Korean Cell Line Bank. The cancer cell line used in our study is not listed in the database of ‘commonly misidentified cell lines maintained by the International Cell Line Authentication Committee (ICLAC). All the cell lines used in our study were authenticated by Short Tandem Repeat (STR) profiling by the cell suppliers and also were tested as free of mycoplasma contamination by the cell suppliers. The cells were maintained in Roswell Park Memorial Institute (RPMI)-1640 medium supplemented with 10% fetal bovine serum (FBS), 100 units/mL penicillin, and 100 μg/mL streptomycin (Invitrogen Co., USA). All culture media and FBS were purchased from Welgene Inc. (Republic of Korea). The cells were incubated at 37 °C in a humidified atmosphere with 5% CO_2_. The cell culture media were routinely changed after every 3 days. The cells were cultured in the customized PDMS clinostat dish. Approximately 2 mL of medium containing an appropriate number of cells was injected through the inlet of the PDMS clinostat dish. After one day of incubation to allow the cells to attach to the bottom of the dish, the cavity of the PDMS clinostat dish was carefully filled fully by adding a medium to prevent bubble formation. The inlet was then sealed with 3 M tape (WT200019360, 3 M Co., USA), and the PDMS clinostat dishes were inserted into the frame. The cells were exposed to SMG (clinorotation) for the indicated hours in the incubator. The cells seeded in the PDMS clinostat dishes and incubated in normal gravity (1 g) were used as control. Images of the cells were captured using a light microscope with the scale bar set to 200 μm. Cells were cultured in six PDMS clinostat dishes, the maximum number that can be inserted on the CS clinostat, to maximize the sample size of the experiment and improve the reliability of the experiment.

### MTT assay

The 3-(4,5-Dimethylthiazol-2-yl)-2,5-Diphenyltetrazolium Bromide (MTT) assay is a rapid colorimetric assay based on the cleavage of the tetrazolium ring of MTT by the dehydrogenases in the active mitochondria of living cells, which provides an estimate of the number of viable cells. MTT reagent (12 mM) was added to the cells and incubated for 3 h by following the manufacturer’s protocol (M6494, Invitrogen Co., USA). At the end of this incubation, 100 µL of dimethyl sulfoxide was added to dissolve the MTT reaction product, formazan, at room temperature for 30 min. Then, cell density was estimated as the absorbance at 540 nm by using an enzyme-linked immunosorbent assay (ELISA) reader (Epoch2, BioTek Co., USA). All cells, including the adherent and floating cells, were collected and used in the MTT assay. The experiment was performed in triplicate and repeated at least thrice.

### Western blot analysis

For Western blot analysis, all cells were lysed for 30 min on ice in a radioimmunoprecipitation assay buffer with a protease inhibitor cocktail (P8340, Sigma-Aldrich Co., USA), and the lysates were cleared by means of centrifugation at 184,000×*g* for 20 min. The proteins were separated on 10% sodium dodecyl sulfate-polyacrylamide gel and electro-transferred onto a polyvinylidene fluoride (PVDF) membrane (#1620177, Bio-R Co., USA). The membrane was then blocked with 3% bovine serum albumin in 1×TBS (Biosesang Co., Republic of Korea) containing 0.1% Tween 20 (Biosesang Co., Republic of Korea) for 4 h and incubated overnight at 4 °C with a 1:500 dilution of primary-antibody-targeting CAV-1 (#3238, Cell Signaling Technology Co., USA). Thereafter, the membranes were rinsed with washing buffer thrice for 15 min each time (1×TBS containing 0.1% Tween 20) and incubated with the appropriate secondary antibody at room temperature for 1 h. After rinsing thrice for 15 min each time with washing buffer at room temperature, the membrane-bound proteins were developed using SuperSignal West Pico PLUS chemiluminescent substrates (Thermo Scientific Co., USA). The protein bands in the Western blot films were quantified using ImageJ software (National Institutes of Health, USA). The experiment was performed in triplicate and repeated at least thrice. β-actin was used as the loading control.

### Statistical analysis

Cell viability was compared between the 1 g and SMG environments by performing a one-way analysis of variance with Turkey’s test for multiple comparisons. All the *p*-values were two-sided, and statistical significance was defined as ^#^*p* < 0.05, and **p < 0.01. Statistical analysis was performed using the Statistical Package for the Social Sciences (version 15.0, SPSS Inc., USA).

### Reporting summary

Further information on research design is available in the Nature Research Reporting Summary linked to this article.

## Results and discussion

### Fabrication results of CS clinostat and PDMS clinostat dish

The CS clinostat was fabricated successfully by 3D-printing all its components separately and then assembling them (Fig. 2a). The key function of the clinostat is to apply rotation to the cells so that they experience gravity evenly in all directions. To realize high-quality 3D rotation with these characteristics, the rotational speeds of the motors driving the two axes must be different^19^. If this is not the case, the cells do not experience full spherical rotation. The more complex the rotation trajectory, the stronger the gravity reduction effect. Therefore, the rotational speeds of the inner- and outer-axis motors were controlled to 4 and 1.8 rpm, respectively; this speed ratio of 4:1.8 was selected based on reference^19^.

The PDMS clinostat dish was designed with the dimensions of 58 mm × 58 mm × 11 mm to ensure it can fit up to six dishes. The aim was to maximize space efficiency considering the limited space available in incubators (Fig. 2b). The advantages of this PDMS clinostat dish were as follows. First, the transparent characteristics of PDMS allowed for easy observation using a microscope. Second, it was easy to fill culture media into the PDMS clinostat dish without causing air bubble formation, which might induce severe mechanical stress in the attached cells and damage them^13^. Culture media and cell suspensions were injected into the dish through a hole with a diameter of 5 mm punched through the top of the PDMS clinostat dish. Air bubbles gathered at this hole owing to buoyancy and were easily removed by pipetting. Lastly, the surface of the PDMS clinostat dish allowed for gas exchange owing to the good permeability of PDMS. It is important to provide the optimal oxygen concentration to the growing cells to maintain viability and functionality^20^. We calculated the amount of oxygen supplied to the cells cultured in the PDMS clinostat dish as follows. The permeation coefficient α of oxygen in the membrane is expressed as follows^21^:

1 $$\alpha =\frac{Vh}{tS\varDelta P}$$

where *V* is the volume of oxygen permeating the membrane (cm^3^), *h* is the membrane thickness (cm), *t* is time (s), *S* is the effective oxygen-permeable area of the membrane (cm^2^), and Δ*P* is the pressure difference between the two sides of the membrane (Pa). The oxygen permeation coefficient *α* of the PDMS membrane was determined to be 3 × 10^−11^ cm^2^ s^−1^ Pa^−1^ according to the reference^22^. The upper membrane thickness of the PDMS clinostat dish *h* was 2 mm, oxygen permeation area *S* was 16.62 cm^2^, and Δ*P* was assumed to be 101.3 kPa. By substituting these values into formula (1), we determined that approximately 28.3 cm^3^ of oxygen was supplied to the PDMS clinostat dish system over 24 h. Because the internal volume of the PDMS clinostat dish was approximately 11.63 cm^3^, we believe that an adequate amount of oxygen was supplied to the system. The benefits of oxygen permeation through the PDMS membrane can alternatively be thought of in terms of the oxygen capacity inside the dish in the absence of oxygen permeation. If there is no external oxygen supply, the cells can only consume the oxygen dissolved in the culture medium. It is theoretically possible to calculate the amount of dissolved oxygen in the volume of the culture media inside the dish. The maximum amount of oxygen that can be dissolved in culture media at 1 atm is 181 µmol/L^23^. Therefore, the culture media inside the current dish, which has a volume of approximately 11.6 mL, can contain up to 2.1 × 10^−6^ mol of oxygen. Because the clinostat is constantly rotating and mixing the media, oxygen gradients within the media need not be considered. The oxygen consumption rate (OCR) of cells ranges from 1 to 120 × 10^−18^ mol cell^−1^ s^−1^ in various cell lines^24^. The OCR of the ovarian cancer cell line (Caov-3) was approximately 30 × 10^−18^ mol cell^−1^ s^−1^ (converted from the OCR value of approximately 1 × 10^−10^ mol/min of 5 × 10^4^ cells in reference)^25^. In our system, cells were initially seeded at 5 × 10^5^ in the clinostat dish. Therefore, the rate at which the cells inside the clinostat dish consumed oxygen was approximately 1.3 × 10^−6^ mol/day. Because the amount of oxygen dissolved in the culture media was 2.1 × 10^−6^ mol, the cells were expected to consume most of the oxygen in the culture media after about 2 days without oxygen supply. When cells are cultured in an enclosed space without oxygen supply, as described above, there is a risk that the cells will be cultured in relative hypoxia, which can affect processes such as cell growth, differentiation, and signaling^23^. Owing to its air permeability characteristics, the PDMS clinostat dish provided a better environment for the cells than that provided by a sealed commercial dish, which does not allow for the permeation of air. In addition, during fabrication, the size of the PDMS clinostat dish can be customized based on experimental requirements, such as the desired number of cells or the number of days of culture, which is another advantage. The cell culture area inside the PDMS clinostat dish measured 46 mm in diameter and 7 mm in height, and it could hold approximately 11.6 mL of medium. This volume of medium was sufficient to allow the cells to survive for more than 4 days without medium change. It was possible to increase the bottom area of the PDMS clinostat dish to up to 70 mm × 70 mm within the current inner frame. The diameter of the cell culture area of this enlarged PDMS clinostat dish was 58 mm (original diameter was 46 mm), and it was possible to increase this area by approximately 60%. If the cells were to be cultured for a longer period (>4 days) without changing the medium, the height of the culture chamber could be customized to hold a larger volume of the medium. If, by contrast, a larger number of cells were to be cultured at once for a short period, the height of the PDMS clinostat dish could be adjusted to mount more PDMS clinostat dishes.

### Mechanical verification of the gravity-reduction effect of CS clinostat

**Fig. 3:**
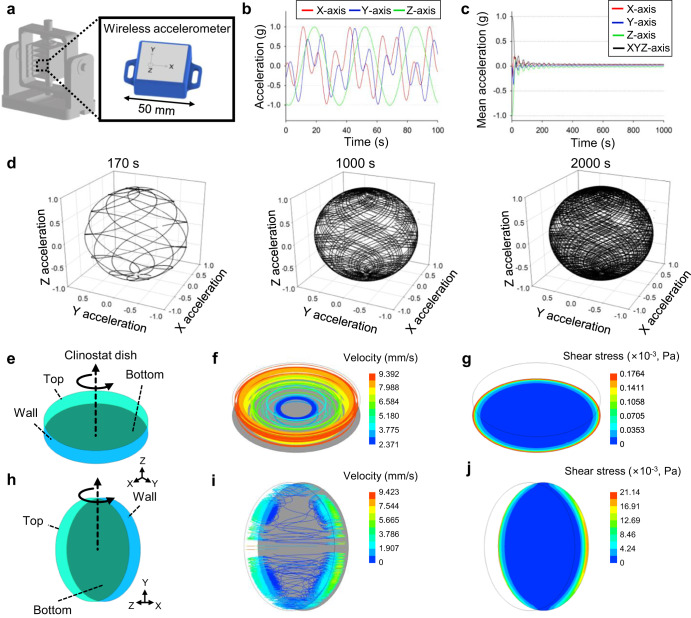
Characterization of CS clinostat and clinostat dish. **a** The wireless acceleration sensor is located at the center of the inner frame of the clinostat for measuring three-axis acceleration. The measured sensor values are transmitted to a smartphone through Bluetooth communication. **b** Acceleration graph over time for each axis. As the clinostat rotated, the accelerations of the *x*, *y*, and *z* axes varied continuously from −1 to 1 g. **c** Mean acceleration graph over time for each axis. Over time, the mean acceleration decreases because acceleration accumulates in all directions. **d** 3D graph showing the distribution of acceleration in the clinostat. After approximately 30 min, the distribution of accelerations appears perfectly spherical, showing that gravity is distributed along all directions inside the clinostat. Computational fluid dynamics simulation of horizontal (**e**–**g**) and vertical (**h**–**j**) rotation of the clinostat dish.

To mechanically verify whether the CS clinostat successfully achieves the SMG environment, the acceleration inside the clinostat was measured using a three-axis accelerometer. Because the internal acceleration of the clinostat changes along the *x*, *y*, and *z* axes, a three-axis accelerometer was used for the acceleration measurements. A sensor that can transmit the axial acceleration value to a mobile phone through Bluetooth communication was attached to the center of the clinostat and rotated (Fig. 3a). As the CS clinostat rotated, the accelerations of the *x*, *y*, and *z* axes varied continuously from −1 to 1 g (Fig. 3b). The mean value of the accumulated acceleration was calculated using the following equation, in which the acceleration of each axis accumulates and decreases with time, which demonstrates the principle of SMG:

2 $${a}_{x,{\rm{mean}}}=\frac{{\sum }_{i=1}^{n}{a}_{x,i}}{n}$$

3 $${a}_{y,{\rm{mean}}}=\frac{{\sum }_{i=1}^{n}{a}_{y,i}}{n}$$

4 $${a}_{z,{\rm{mean}}}=\frac{{\sum }_{i=1}^{n}{a}_{z,i}}{n}$$

5 $${a}_{{\rm{mean}}}=\sqrt{{{a}_{x,{\rm{mean}}}}^{2}+{{a}_{y,{\rm{mean}}}}^{2}+{{a}_{z,{\rm{mean}}}}^{2}}$$

where *a*_*x*,*i*_, *a*_*y*,*i*_, and *a*_*z*,*i*_ are the acceleration values of the three axes; *n* is the total number of measured accelerations; *a*_*x*,*mean*_, *a*_*y*,*mean*_, and *a*_*z*,*mean*_ are the mean acceleration values of the three axes; and *a*_*mean*_ is the sum of the mean acceleration values of each axis. The mean acceleration of each axis accumulated as time passed and then gradually decreased (Fig. 3c). The results of the sensor experiment confirmed that the cells remained under the influence of gravity, but because the gravity vector continued to change with time due to rotation, the mean acceleration vector decreased to about 3 × 10^−2^ g within 1 h and continued to decrease. To quantify the acceleration value, the mean acceleration within one rotation period of the CS clinostat was calculated. Because the rotation speeds of the two axes were different (1.8 and 4 rpm, respectively), it was difficult to define a period. For this reason, approximately 33 s, the time required for one rotation of the *z*-axis, was defined as the period. The measured mean accelerations of the *x*, y, and z axes were 0.00122, −0.0356, and −5.75 × 10^−5^ g during the 0–33 s period; 0.0692, −0.0334, and −9.41 × 10^−^^5^ g during the 33–66 s period; and 0.0940, 0.0336, and 6.809 × 10^−5^ g during the 66–99 s period. Moreover, the results confirmed that a time-averaged SMG environment was created inside the CS clinostat. It has been reported that the closer the three-dimensional rotational acceleration trajectory to a perfectly spherical shape, the more successful is the simulation of a microgravity environment^19^. Therefore, the distribution of accelerations was confirmed by displaying the acceleration values of each of the axes in a three-dimensional graph (Fig. 3d). Over a short period, the distribution of accelerations did not exhibit a perfectly spherical shape, but because the rotational speeds of two axes were different, the distribution of accelerations increasingly resembled a spherical shape as time progressed. After approximately 30 min, an almost perfect sphere was observed. Based on this result, we concluded that the cells inside the CS clinostat were subjected to reduced gravity in all three-dimensional directions.

Notably, the smaller the rotational radius of the PDMS clinostat dish, the smaller the magnitude of the centrifugal acceleration, which minimizes the exposure of the cells to unwanted centrifugal forces. The centrifugal acceleration applied to the cells was linearly proportional to the square of the rotational speed and distance from the rotational center. The CS clinostat system was designed to culture as many cells as possible in a compact space, such that the centrifugal acceleration acting on the cells was minimized. The distance from the center of rotation to the furthest cell of the CS clinostat system was 39.0 mm, which was approximately 39% of the distance reported for another clinostat system^10^, where the maximum distance to the cell was 99.2 mm. This means that the cells in the CS clinostat system experienced up to 39% lower centrifugal force.

However, a flow of medium was inevitably generated in the PDMS clinostat dish during the operation of the CS clinostat. We simulated the flow driven by the rotation of the dish by using ANSYS Fluent 19.2 (ANSYS Inc., USA), a commercial computational fluid dynamic (CFD) tool. By using the CFD model, a steady-state simulation was performed under the rotational condition of 4 rpm in a computational domain composed of hexahedral meshes with 378,200 grids (Supplementary Fig. 4); the rotational axes were horizontally (Fig. 3e) and vertically (Fig. 3h) aligned to the CS clinostat base. The working fluid inside the PDMS clinostat dish was assumed to be water. Owing to the wall effect of the rotating dish, a flow was formed, and it induced shear stress at the bottom of the dish. At both the horizontal and vertical positions, on the bottom area near the wall, a relatively high shear stress was induced compared to that at the center of the bottom because flow mainly occurred near the wall of the dish (Fig. 3f, i). On the bottom of the dish, the maximum shear stresses were calculated to be 0.42 × 10^−3^ Pa and 19.16 × 10^−3^ Pa under horizontal axis rotation and vertical axis rotation, respectively. Notably, it was predicted that most of the area on the bottom of the dish would be exposed to a shear stress of less than 2.11 × 10^−^^3^ Pa (Fig. 3g, j). This simulated shear stress in the PDMS clinostat dish was less than the shear stress of 10^−2^ Pa reported in the interstitial tissue of the human body^26,27^. Therefore, we conclude that this rotation condition is suitable for performing cell-culture-based research. To verify the reliability of the simulation, a convergence study of the maximum wall shear stress was conducted by varying the number of grids (Supplementary Fig. 5). The simulation results obtained using 160,365 grids, approximately 57% less than the original 378,200 grids, showed a difference of approximately 33.44% in the maximum wall shear stress. However, when the number of grids was 505,655, about 33% more than the original number of grids, the maximum wall shear stress showed a small difference of approximately 9.05%. Therefore, we concluded that the simulation results obtained using the original number of grids (378,200) could be reliable.

### Vibration measurements

**Fig. 4:**
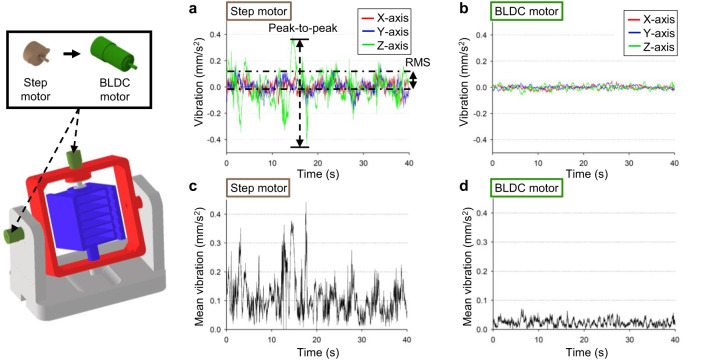
Vibration measurement results of fabricated clinostat comprising step motor and BLDC motor. Vibration graph of each axis of the step motor clinostat (**a**) and BLDC motor clinostat (**b**) as a function of time. The peak-to-peak vibration values of the step motor clinostat and BLDC clinostat are 0.814 and 0.114 mm/s^2^, respectively, and the corresponding root mean square (RMS) values are 0.124 and 0.026 mm/s^2^. Mean vibration graph of the step motor clinostat (**c**) and BLDC motor clinostat (**d**) as a function of time. The measured maximum values of the mean vibration of the step motor clinostat and BLDC clinostat are 0.442 and 0.070 mm/s^2^, respectively.

The vibrations generated by the CS clinostat motors should be minimized because they can have undesirable effects on cells. The vibration generated in the CS clinostat was measured using a three-axis accelerometer. Because the CS clinostat was fabricated using a 3D printer, there was a limit to reducing vibration through precise manufacturing. However, it was confirmed that the amount of vibration generated differed depending on the type of motor used in the clinostat. To quantitatively analyze the clinostat vibrations according to the type of motor used, the vibrations of the CS clinostat fabricated with a step motor (STH-36C1055, Shinano Kenshi Co., Japan) and a BLDC motor (BL2419A, D&J WITH Co., Republic of Korea) were measured and compared. An accelerometer was placed on the CS clinostat base for vibration measurement. The measured vibration values of the CS clinostat along the *x*, *y*, and *z* axes are expressed as graphs in Fig. 4a, b. The peak-to-peak value, which is the difference between the maximum and minimum values of vibration, was measured as 0.814 and 0.114 mm/s^2^ for the step motor clinostat and the BLDC motor clinostat, respectively. The corresponding root mean square (RMS) vibration values were 0.124 and 0.026 mm/s^2^. The peak-to-peak and RMS vibration values of the BLDC motor CS clinostat were approximately 86% and 79% lower than those of the step motor CS clinostat, respectively. In terms of the mean vibration value, which represents the sum of the vibration values along the *x*, *y*, and *z* axes (Fig. 4c, d), the maximum mean vibration value was 0.070 mm/s^2^ for the BLDC motor CS clinostat, which was approximately 84% lower than the value of 0.442 mm/s^2^ for the step motor CS clinostat. Therefore, we fabricated the CS clinostat by using the BLDC motor to reduce unwanted vibration effects on the cells inside it.

### Mechanical characteristics of the clinostat

**Fig. 5:**
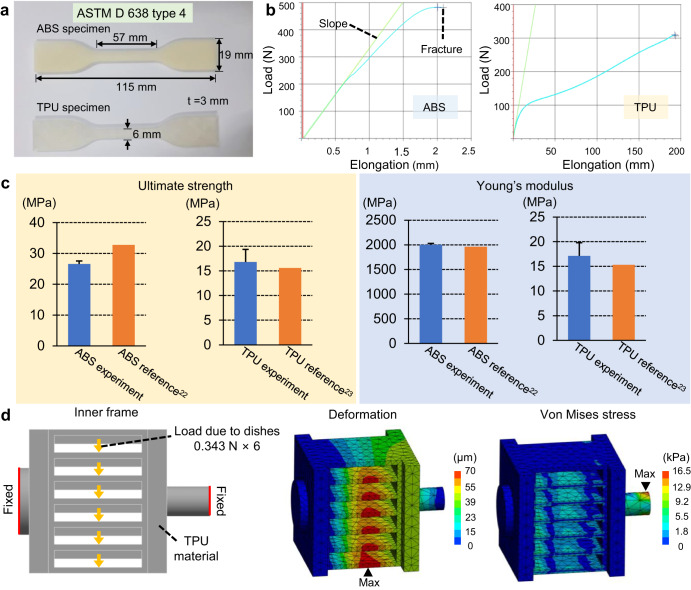
Tensile tests and static structural simulations for verifying mechanical stability of CS clinostat. **a** ABS and TPU specimens are used in the tensile tests. The specimen is 3D-printed with the ASTM D 638 type four standard shape. **b** Graph of tensile test results of ABS and TPU. The blue line indicates the data points of the tensile test, and the green line indicates the slope in the elastic region. **c** The ultimate strength and Young’s modulus of ABS are 26.55 ± 1.00 MPa and 2003.33 ± 24.50 MPa, respectively. The ultimate strength and Young’s modulus of TPU are 16.79 ± 2.5 MPa and 17.13 ± 2.68 MPa, respectively. **d** Static structural simulations of the inner frame made of TPU material. The six dishes in each slot exert a downward force of 0.343 N. The maximum deformation and von Mises stress are 70.76 µm and 0.0165 MPa, respectively. All error bars represent standard deviation.

To confirm the mechanical stability of the 3D-printed CS clinostat, a tensile test, and a static structural simulation were performed (Fig. 5). Unlike other clinostats made of tough materials, such as aluminum or iron, the CS clinostat device was 3D- printed using a relatively low-strength material to realize easy fabrication, reduce cost, and increase accessibility. Therefore, it was necessary to verify that the CS clinostat was mechanically stable. First, to investigate the mechanical properties of the 3D printer material, ABS and TPU specimens for tensile testing were 3D-printed according to the ASTM D 638 type 4 standard (Fig. 5a). Owing to the characteristics of the 3D printer, the mechanical properties of the printed shape can vary depending on the printing conditions. If the mechanical properties of the material itself are applied, the stability of the system may be exaggerated. Therefore, because the mechanical properties of the 3D-printed material were required, rather than those of the material itself, the specimens were printed under the same conditions as those in the CS clinostat (Supplementary Fig. 1), and tensile tests were performed. The elongation caused by applying loads to the ABS and TPU specimens by using a tension–compression tester is shown in Fig. 5b. The blue line indicates the data points of the tensile test, and the green line indicates the slope in the elastic region. The reference and experimental values of ultimate strength and Young’s modulus are summarized in Table 1 and Fig. 5c. The ultimate strength of ABS was 26.55 ± 1.00 MPa, and its Young’s modulus was 2003.33 ± 24.50 MPa. The ultimate strength of TPU was 16.79 ± 2.55 MPa, and its Young’s modulus was 17.13 ± 2.68 MPa. The ultimate strength and Young’s modulus obtained in the experiment were almost the same as those of the standard, with a difference of approximately 20%^28,29^. The strength and Young’s modulus of ABS were approximately 36% higher and 120 times greater than those of TPU, respectively, while the elasticity of TPU was approximately 90 times higher than that of ABS. Therefore, in the CS clinostat, for overall durability, the base and outer frame were made of high-rigidity ABS material, and the inner frame was made of high-elasticity TPU material to ensure that the PDMS clinostat dishes could be inserted easily. Static structural simulations of the inner frame made of TPU were performed by applying the properties obtained from the tensile test (Fig. 5d). Because one PDMS clinostat dish containing medium weighed approximately 35 g, six PDMS clinostat dishes applied a force of 2.058 N on the inner frame. The simulation results indicated that the maximum deformation of the inner frame was 70.766 µm in its lowermost part. Because the distance between two layers in the inner frame was 11 mm, the deformation of 70.766 µm due to the stress acting on the PDMS clinostat dishes, which was only approximately 0.6% of 11 mm, did not affect this system. The maximum von Mises stress was 0.0165 MPa, which was only 0.1% of the experimentally measured maximum stress of 16.79 MPa. In this study, we confirmed that the CS clinostat system was mechanical by conducting tensile tests and static structural simulations. Because the weight of the 3D printed material was significantly lower than that of conventional metallic materials such as iron and aluminum, a considerably lower motor torque was required to operate this lightweight CS clinostat. This allows the CS clinostat to operate using smaller motors and reduces its overall weight and size.

**Table 1:** Tensile test results of ABS and TPU obtained in this study and those reported in the literature.

	ABS experiment	ABS reference^28^	TPU experiment	TPU reference^29^
Ultimate stress (MPa)	26.55 ± 1.00	32.8	16.79 ± 2.55	15.6
Young’s modulus (MPa)	2003.33 ± 24.50	1960	17.13 ± 2.68	15.3

### Validation of reducing gravity effect on cells

**Fig. 6:**
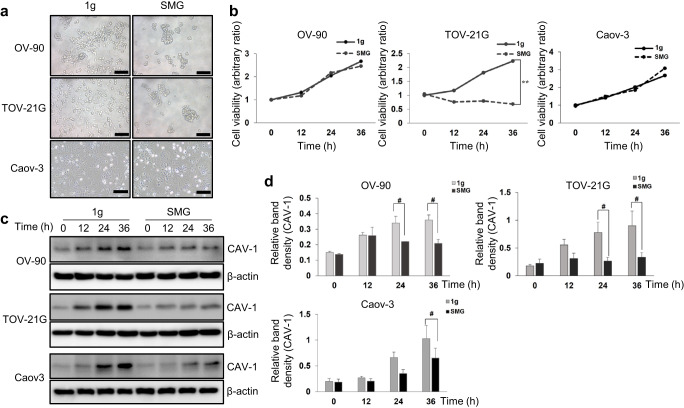
Confirmation of SMG condition generated using CS clinostat. **a** Approximately 5 × 10^5^ of the cells are seeded in the PDMS clinostat dish and incubated in 1 g for 24 h. These cells are then moved into the CS clinostat and incubated for 12, 24, and 36 h. Cells cultured in 1 g are used as controls. Microscopic images show the cells on the PDMS clinostat dish after 24 h of rotation (Magnification: 100×, scale bar: 200 μm). **b** Cell viability is measured using an MTT assay. The arbitrary ratio is calculated as the ratio of the OD540 value of the SMG group relative to that of the control group (1 g at 0 h) at each time point (**p < 0.01). **c** Total protein is extracted, and CAV-1 and SMG marker expression is visualized by means of western blot analysis with β-actin as a loading control. CAV-1 is significantly lowered in the cells cultured in the CS clinostat compared to that of the cells cultured in 1 g, indicating successful generation of SMG. **d** The intensities of the CAV-1 bands are normalized against that of β-actin for comparison (^#^*p* < 0.05). The data were analyzed by using the ANOVA with Turkey’s test for multiple comparisons. All error bars represent standard deviation.

To validate the performance of the clinostat and the realization of the SMG effect on the cells, we incubated the three ovarian cancer cell lines in the CS clinostat. We observed visible changes and compared the results with those of the cells incubated under 1 g conditions. The OV-90 and TOV-21G cells aggregated to form multiple spheroids in the SMG environment, whereas they spread out as they multiplied in the 1 g environment (Fig. 6a). This finding can be attributed to several reasons. First, changes in the cytoskeletal proteins, which are well-known targets of exposure to real microgravity, might change cell morphology and cause aggregation^30^. Second, changes to the adhesion molecules because of SMG might contribute to spheroid formation^30^. Third, both real and SMG neutralize sedimentation, and this forces 3D growth^31^.

Interestingly, the cells responded differently to SMG. Caov-3 did not exhibit spheroid formation, unlike OV-90 and TOV- 21 G. The viability of the OV-90 and Caov-3 cells did not differ between the two gravity conditions, while the viability of the TOV-21G cells decreased markedly in the SMG environment (Fig. 6b). Real and SMG altered the physiological functions of the cells by altering intracellular signaling and gene expression^32^. The normal cells adapted relatively well to reduced gravity, whereas the cancer cells harbored various genetic abnormalities, and therefore, all cells might not be able to tolerate reduced gravity. Different genetic alterations in each of the types of cells may explain their different reactions to changes in gravity. Further studies are needed to elucidate why the cancer cells were affected differently by the SMG environment, and a comparison with the results of experiments conducted in real microgravity is needed as well.

Additionally, the protein expression of CAV-1, which is known to decrease when cells are exposed to reduced gravity in space and, therefore, serves as a surrogate marker of SMG^16^, was significantly downregulated in the cells incubated in the CS clinostat, indicating successful SMG generation (Fig. 6c, d). Taken together, our findings indicated that biologically, the CS clinostat system successfully generated an SMG environment, which had various effects on different types of cells.

The OV-90 cells were cultured at the rotational speeds of 20 and 10 rpm to verify the effects of high rotational speed. The optimal rotational speed of the clinostat for all types of cells is yet to be determined. Previous studies have implemented various rotational speeds for various types of cells (human embryonic kidney (HEK293) cells, normal human fibroblast (1BR-hTERT) cells)^33,34^. We found that the OV-90 cells were detached from the coated bottom at the clinostat rotation speed of 20 rpm but not at speeds less than 10 rpm. The peak centrifugal acceleration (*a*_*pc*_) in a clinostat with two rotating axes can be expressed as follows^13^:

6 $${a}_{pc}[{\rm{m}}/{s}^{2}]=2.41\,{\omega }^{2}{r}_{m}$$

where *ω* is the angular velocity (rad/s), and *r*_*m*_ is the maximum radius from the rotation center (m), which is 0.039 m in our system. The *a*_*pc*_ values at the rotation speeds of 20 and 10 rpm were 0.41 m/s^2^ (0.04 g) and 0.10 m/s^2^ (0.01 g), respectively. The acceleration at 20 rpm seemed harsh for the OV-90 cells, and therefore, a slower rotation speed was recommended for the OV-90 cells. The rotational speeds of our system were 4 and 1.8 rpm along the two axes, which were considerably slower than 20 rpm. Therefore, the cells in our system were not damaged because of centrifugal acceleration.

### Supplementary information

Reporting Summary

supplementary information

## Data Availability

Data supporting this research are publicly available. The results of acceleration and vibration measurements of the clinostat, mechanical property experiments of 3D printed materials, and band density and MTT data of cell experiments are available from Figshare.
