# Supplementary material for: Customized small-sized clinostat using 3D printing and gas-permeable polydimethylsiloxane culture dish
Source: NPJ Microgravity. 2023 Aug 11;9:63. doi: 10.1038/s41526-023-00311-1 (PMC10421914; doi:10.1038/s41526-023-00311-1)
Supplement: Supplementary file 2 — supplementary information [file 41526_2023_311_MOESM2_ESM.pdf]

Supplementary Information

Customized small-sized clinostat using 3D printing and gas permeable polydimethylsiloxane culture dish

Daehan Kim[1], Que Thanh Thanh Nguyen[2], Seungjin Lee[1], Kyung-Mi Choi[2], Eun-Ju Lee[2*] and Joong Yull Park[1,3*]

[1] Department of Mechanical Engineering, Graduate School, Chung-Ang University, Seoul 06974, Republic of Korea

[2] Department of Obstetrics and Gynecology, School of Medicine, Chung-Ang University, Seoul 06974, Republic of Korea

[3] Department of Intelligent Energy and Industry, Graduate School, Chung-Ang University, Seoul 06974, Republic of Korea

Supplementary Figures:

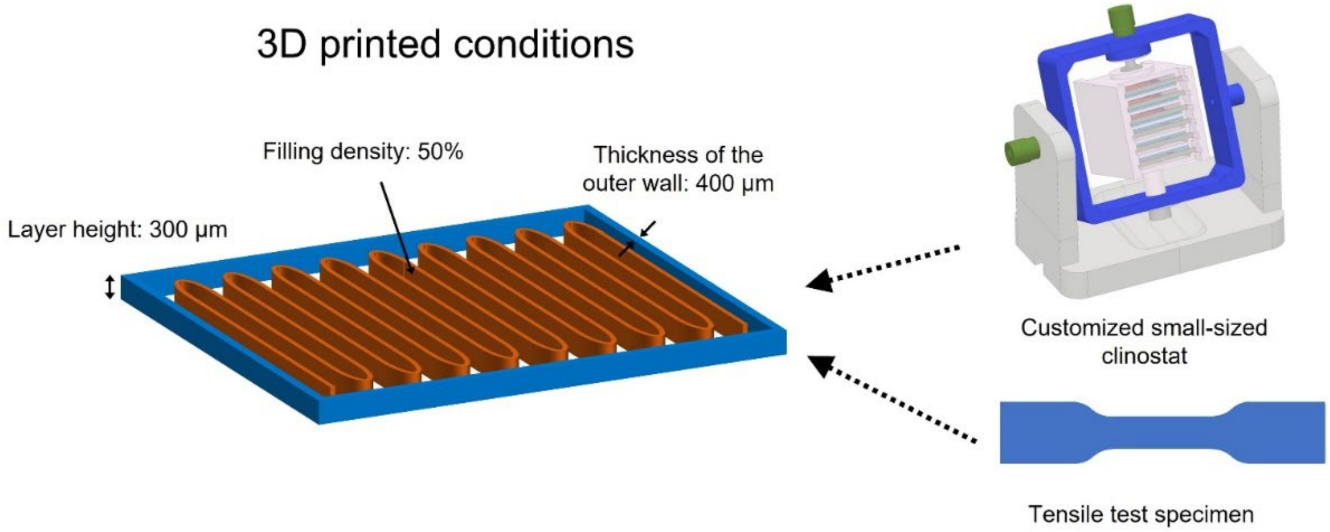

Supplementary Figure 1. 3D printed conditions of customized small (CS) clinostat and tensile test specimen. The 3D printer layer height was 300 μm, the printed outer wall thickness was 400 μm, and the infill density was 50% (; note that typical infill density ranges 15 to 50%)

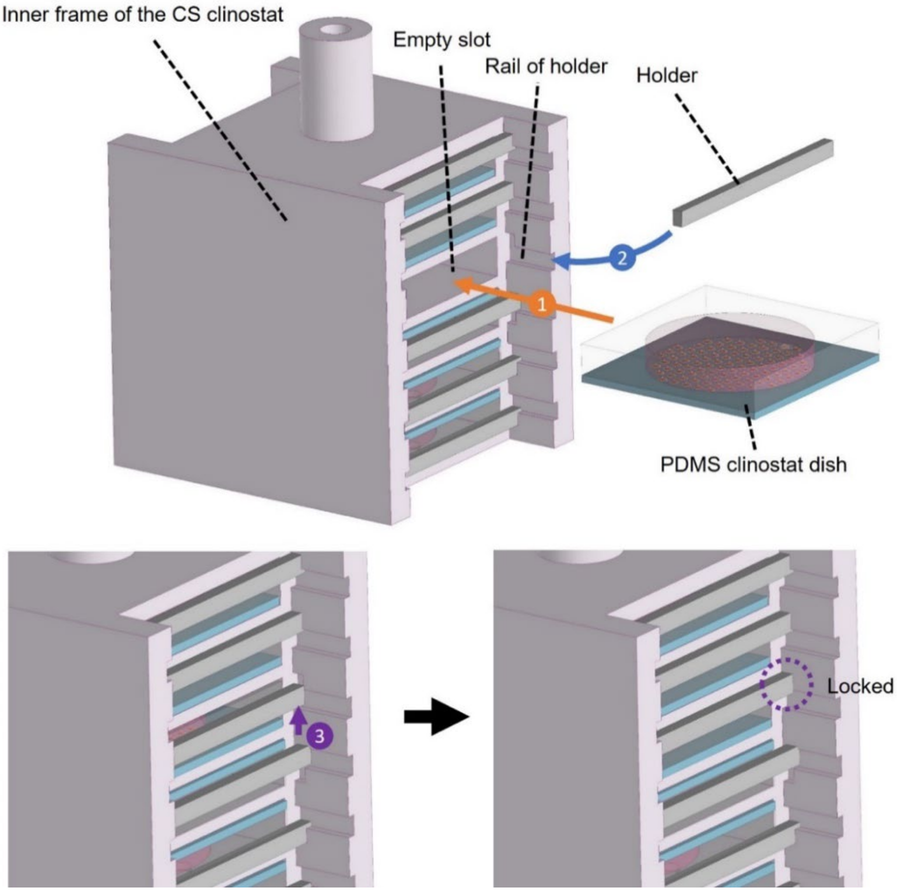

Supplementary Figure 2. Sequence of inserting the PDMS clinostat dish into the inner frame of the CS clinostat. Firstly, the PDMS clinostat dish is inserted into the empty slot of the inner frame. Secondly, the holder is inserted through a rail of the holder. Thirdly, the holder is secured by pushing up slightly at the end of the rail. The holder prevents falling of the PDMS clinostat dish during the rotation of the CS clinostat without a tape.

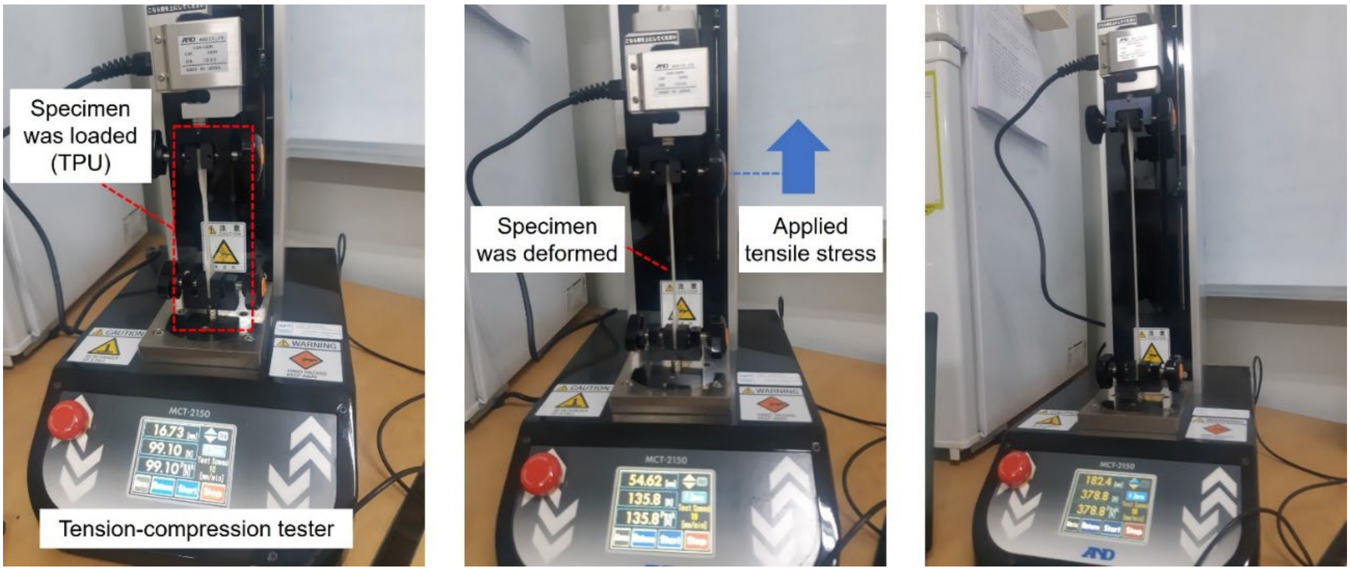

Supplementary Figure 3. Tensile test process of 3D printed specimen. The 3D printed specimen (TPU or ABS) was placed in a tension-compression tester (MCT-2150, A&D Co., Japan). Bone-shaped ABS and TPU were printed in conformity with ASTM D 638 type 4, an international standard for tensile testing. The specimen was tensioned at a rate of 10 mm/min.

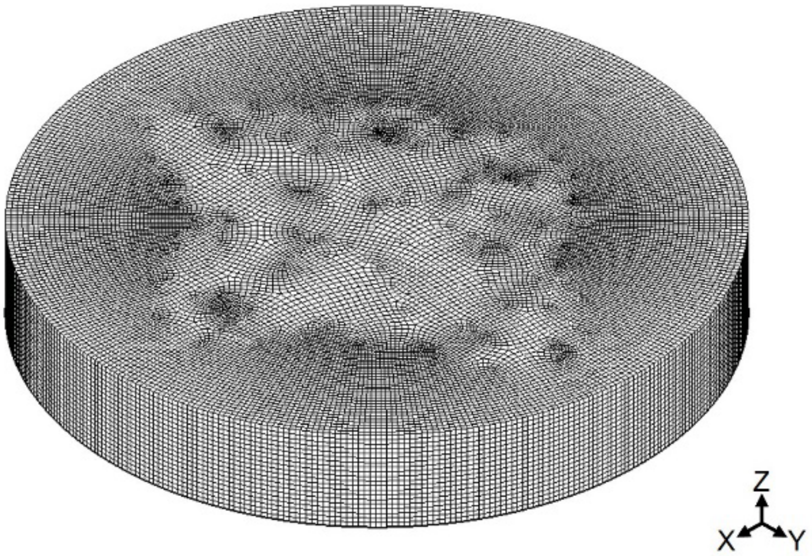

Supplementary Figure 4. A mesh of the computational fluid dynamics simulation of internal flow of a clinostat dish. A cell culture area inside the clinostat dish was cylindrical, 46 mm in diameter and 7 mm in height. In the CFD model, the steady-state simulation was performed under the rotational condition of 4 rpm of the computational domain with the hexahedral mesh and the rotational axis horizontally and vertically aligned to the CS clinostat base, respectively, and the water conditions was assumed as the working fluid of clinostat dish. The flow in the clinostat dish was calculated using the following governing equations for continuity (Eq. 1) and momentum (Eq. 2) (Ref: Fluent, A. I. 17.0 ANSYS Fluent Theory Guide. Canonsburg (PA): ANSYS, 2016).

$$\frac{\partial \rho}{\partial t} + \nabla \cdot (\rho u) = S_m \qquad \text{(Eq. 1)}$$

$$\frac{\partial (\rho u)}{\partial t} + \nabla \cdot (\rho u u) = -\nabla \rho + \nabla \cdot (\tau) + \rho g + F \qquad \text{(Eq. 2)}$$

where ρ is the density of the working fluid, u is the velocity component, S_m is the mass added from the source, τ is the stress component, g is the gravitational acceleration, and F is the force component. To predict the turbulence phenomenon, the standard k-ε model was applied. The following transport equations were used in the standard k-ε model to calculate the turbulent kinetic energy k (Eq. 3) and its dissipation rate ε (Eq. 4).

$$\frac{\partial (\rho k)}{\partial t} + \frac{\partial (\rho k u_i)}{\partial x_i} = \frac{\partial}{\partial x_j}\left[\left(\mu + \frac{\mu_t}{\sigma_k}\right)\frac{\partial k}{\partial x_j}\right] + G_k + G_b - \rho\varepsilon - Y_M \qquad \text{(Eq. 3)}$$

$$\frac{\partial (\rho \varepsilon)}{\partial t} + \frac{\partial (\rho \varepsilon u_i)}{\partial x_i} = \frac{\partial}{\partial x_j}\left[\left(\mu + \frac{\mu_t}{\sigma_\varepsilon}\right)\frac{\partial \varepsilon}{\partial x_j}\right] + C_{1\varepsilon}\frac{\varepsilon}{k}(G_k + C_{3\varepsilon}C_b) - C_{2\varepsilon}\rho\frac{\varepsilon^2}{k} \qquad \text{(Eq. 4)}$$

where G_k and G_b are meaning the generation of turbulence kinetic energy by the mean velocity gradients and buoyancy, respectively, and Y_M is the contribution of the fluctuating dilation in compressible turbulence of the overall dissipation rate. μ is the dynamic viscosity and μ_t is the turbulent (or eddy) viscosity. $C_{1\varepsilon}$, $C_{2\varepsilon}$, and $C_{3\varepsilon}$ are constants, and σ_k and σ_ε are the turbulent Prandtl numbers of the kinetic energy and the dissipation rate, respectively.

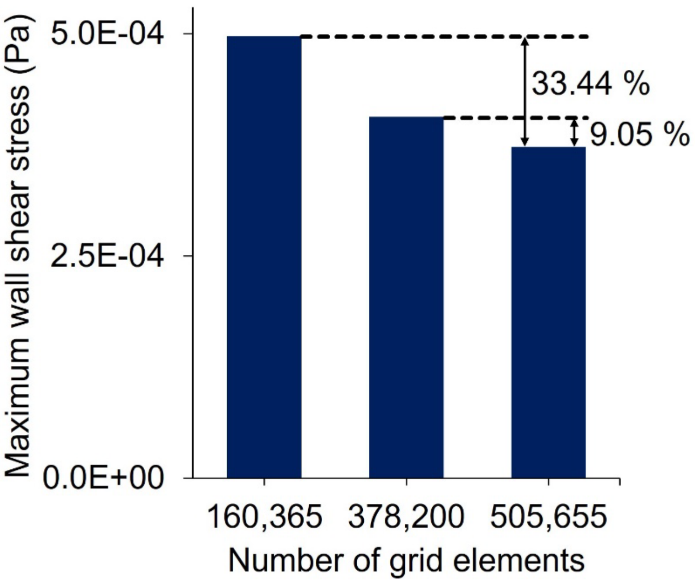

Number of elements	Max wall shear stress (Pa)	difference (%)
160,365	0.000497671	33.44496851
378,200	0.000406685	9.048079991
505,655	0.000372941	

Supplementary Figure 5. Grid independence test results. The maximum wall shear stress was calculated at the bottom of the dish in each test case, and the results were compared. The maximum wall shear stress of the increased grid (505,655) was 33.44% different from the maximum wall shear stress of the reduced grid (160,365), however it was only 9.05% different from the maximum wall shear stress of the original grid (378,200). Therefore, the simulation results of the original grid were considered reliable.
